# Linkage Analysis of Genomic Regions Contributing to the Expression of Type 1 Diabetes Microvascular Complications and Interaction with HLA

**DOI:** 10.1155/2015/694107

**Published:** 2015-10-11

**Authors:** Ettie M. Lipner, Yaron Tomer, Janelle A. Noble, Maria C. Monti, John T. Lonsdale, Barbara Corso, David A. Greenberg

**Affiliations:** ^1^Integrated Center for Genes, Environment and Health, National Jewish Health, Denver, CO 80206, USA; ^2^Department of Pharmacology, University of Colorado Denver School of Medicine, Aurora, CO 80045, USA; ^3^Department of Medicine, Mount Sinai Medical Center, New York, NY 10013, USA; ^4^Children's Hospital Oakland Research Institute, Oakland, CA 94702, USA; ^5^National Research Council, Neuroscience Institute, 35128 Padova, Italy; ^6^National Disease Research Interchange, Philadelphia, PA 19103, USA; ^7^Battelle Center for Mathematical Medicine, Nationwide Children's Hospital, Columbus, OH 43215, USA; ^8^Department of Pediatrics, Wexner Medical Center, Ohio State University, Columbus, OH 43205, USA

## Abstract

We conducted linkage analysis to follow up earlier work on microvascular complications of type 1 diabetes (T1D). We analyzed 415 families (2,008 individuals) previously genotyped for 402 SNP markers spanning chromosome 6. We did linkage analysis for the phenotypes of retinopathy and nephropathy. For retinopathy, two linkage peaks were mapped: one located at the HLA region and another novel locus telomeric to HLA. For nephropathy, a linkage peak centromeric to HLA was mapped, but the linkage peak telomeric to HLA seen in retinopathy was absent. Because of the strong association of T1D with DRB1^*^03:01 and DRB1^*^04:01, we stratified our analyses based on families whose probands were positive for DRB1^*^03:01 or DRB1^*^04:01. When analyzing the DRB1^*^03:01-positive retinopathy families, in addition to the novel telomeric locus, one centromeric to HLA was identified at the same location as the nephropathy peak. When we stratified on DRB1^*^04:01-positive families, the HLA telomeric peak strengthened but the centromeric peak disappeared. Our findings showed that HLA and non-HLA loci on chromosome 6 are involved in T1D complications' expression. While the HLA region is a major contributor to the expression of T1D, our results suggest an interaction between specific HLA alleles and other loci that influence complications' expression.

## 1. Introduction

Retinopathy, nephropathy, and neuropathy are chronic microvascular complications responsible for much of the morbidity and mortality in type 1 diabetes (T1D). Evidence for familiarity in complications has been clearly demonstrated, suggesting a genetic contribution to these phenotypes [1–4]. Although numerous linkage and association studies have focused on identifying T1D susceptibility loci, there has been little analysis of genetic influences on complications. In the few linkage analyses that focused on identifying T1D-related complications of susceptibility loci, only nephropathy has been investigated [5–9]. To our knowledge, there have been no* linkage* studies aiming at investigating the influence of the HLA region on the expression of complications.

Therefore, the aim of our study was to use the robust method of linkage analysis in a large well-characterized cohort of T1D families to identify gene-loci that predispose to type 1 diabetic complications. We focused our genome analysis on chromosome 6 to follow up our previous work showing the importance of loci on chromosome 6 to the genetic predisposition of T1D complications [10].

## 2. Methods

### 2.1. Family Recruitment and Data Collection

Families were ascertained through the presence of at least one family member with type 1 diabetes. A questionnaire was given to the proband or parents as well as to additional family members. The questionnaire included demographic, medical, genealogical, and familial information about T1D as well as complications. More details can be found in Lipner et al. [10]. Other information to assure the accuracy of participants' disease status is discussed below.

### 2.2. HBDI Data

Our dataset included 415 families (2,008 individuals) with T1D cases diagnosed before age 30 (Tables 1 and 2). Female percentage was 49%. 239 individuals in 159 families had at least 1 microvascular complication: 219 individuals had retinopathy, 87 had nephropathy, and 76 had neuropathy. Some subjects had more than one complication.

### 2.3. Assessment of Diabetes and Diabetic Complications

The accuracy of the self-reported information about complications was evaluated by the following.

1. Including extra questions about complications-related conditions in the questionnaire. Reports of macular edema, vitrectomy, or complete or partial blindness were considered an indicator of retinopathy; reports of end-stage renal failure, kidney failure, or repeated high urinary albumin levels were considered an indicator of nephropathy. In cases of inconsistencies (e.g., report of macular edema but not retinopathy), further investigations were carried out through phone interviews. In order to avoid ambiguity, only the most obvious or severe cases of retinopathy or nephropathy were classified as “affected.”
2. Data available from follow-up were used to confirm or update the presence/absence and progression of complications.
3. 179 patients had medical records available allowing us to verify phenotype according to American Diabetes Association guidelines [11–14].
4. Information indicating* absence* of a complication in a family member with T1D was considered reliable only if the subject was without that complication for at least 15 years after type 1 diabetes onset.

### 2.4. Assessment of Self-Reported Diabetic Complications

The accuracy of self-reported information was assessed in three ways. (1) Additional questions were included in questionnaires given to both patients and family members. (2) Follow-up telephone interviews were carried out by HBDI staff if the questionnaire was unclear. (3) Medical records were assessed on T1D patients that submitted medical records with the questionnaire (179 (2.3%)). (4) Follow-up questionnaires went to a subset of families for updated information about the development of complications, new cases of diabetes, and other related medical history. Twenty-three percent of the type 1 diabetics in the HBDI database responded with follow-up data and 10% of subjects included medical records with the questionnaire. On-going validation at HBDI has shown that questionnaire answers accurately reflect physician diagnosis in the medical records [1]. Thus, the severity of reported symptoms, corroboration of accuracy using patients' medical records, and follow-up contact with a sample of patients and families assure phenotype accuracy.

Since the majority of patients' diagnoses are self-reported, T2D may have occasionally been misclassified as T1D. The presence of autoantibodies would confirm an autoimmune response. Autoantibody markers from a random sample of T1D study subjects (*n* = 76) characterized study sample homogeneity. Only 5% of T1D-classified patients in this subsample tested negative for autoantibodies. Also, the absence of autoantibodies is not proof of misdiagnosis. 3.5%–10% of T1D patients are autoantibody-negative [15, 16]. Thus, misdiagnosis of T2D as T1D is unlikely to have affected our results.

Reliability of self-report questionnaires: self-reports of diabetes have demonstrated excellent agreement with the use of medical records [17]. Further, other studies have shown that self-reporting of diabetes tends to be more accurate than other chronic disease self-reports [18, 19]. We did not identify any studies comparing the use of medical records with self-report of diabetic microvascular complications.

Thus, if any T1D families were actually T2D, or if some patients with complications were misdiagnosed as complications-free, it is unlikely to have introduced bias into our results for two major reasons. (1) Misdiagnosing an affected person as “unaffected” decreases linkage evidence but does not lead to false linkage evidence [20]; it introduces reduced penetrance, which the analysis takes account of through the penetrance parameter. (2) Classifying a truly unaffected person as “affected” has the effect of severely reducing the evidence for linkage but can be taken into account via the HLOD parameter. Strong evidence of linkage, with high LOD and HLOD scores, does not eliminate the possibility of misdiagnosis or heterogeneity but finding false evidence of linkage* because* of heterogeneity or misdiagnosis is highly unlikely, since any misdiagnosis degrades the linkage signal.

### 2.5. Genotyping

The Center for Inherited Disease Research (CIDR) at the National Human Genome Research Institute did the genotyping. Average marker spacing was 0.58 cM. We restricted our analyses to the 402 marker SNPs on chromosome 6.

### 2.6. Phenotype Definitions

“Affected” phenotypes were (1) the presence of any microvascular complication, (2) the presence of retinopathy, and (3) the presence of nephropathy. Each phenotype was analyzed separately. The neuropathy phenotype yielded too little linkage information and no further analyses were done using that phenotype. T1D patients without complications were classified as “unaffected.” Individuals without T1D were excluded from these analyses (except parents). Families had at least one “affected” and one “unaffected” family member, or at least two affected members (e.g., at least two siblings with T1D, at least one of whom had complications).

### 2.7. Linkage Analysis

Multipoint LOD (“logarithm of odds”) scores and heterogeneity LOD scores (HLOD scores) were calculated using the GeneHunter program [21]. The HLOD reflects the evidence for linkage taking into account possible heterogeneity within the dataset; that is, only a proportion of families in the dataset are linked to the marker. We assumed both dominant and recessive modes of inheritance [22]. A dominant gene frequency of 0.1 and a recessive gene frequency of 0.2 were assumed. Preliminary analyses assuming three levels of penetrance (90%, 50%, and 25%) and a dominant and recessive mode of inheritance showed that a penetrance of 25% and recessive inheritance yielded the highest LOD scores [20, 21, 23, 24]. Subsequently, for all reported analyses, we assumed a recessive mode of inheritance and 25% penetrance. In all calculations, if assuming a recessive mode of inheritance led to positive LOD scores, so did assuming a dominant inheritance, indicating evidence in favor of linkage irrespective of assumed mode of inheritance. Almost entirely, the LOD score assuming a recessive inheritance model was notably higher than dominant, so the LOD and HLOD assuming recessive inheritance are the scores we report.

We performed preliminary analyses on the phenotype “any complication” but our subsequent analysis classified only subjects with retinopathy (RET) as “affected” and, separately, only subjects with nephropathy (NEPH) as “affected.”

### 2.8. Stratification on the Presence of T1D High-Risk HLA Alleles in the Proband

We previously showed [10] that the DRB1^*^03:01 allele provided a protective effect against retinopathy. Therefore, we explored the influence of DRB1^*^03:01 or DRB1^*^04:01 on the linkage evidence in subsets of families grouped by the presence of DRB1^*^03:01 or DRB1^*^04:01 in the proband. Our aim in stratifying was to identify possible gene-gene interaction between the novel loci we identified and these HLA alleles, since we had previously identified the alleles' differential effect on complications risk [10]. We also carried out “pure” DRB1^*^03:01 or DRB1^*^04:01 analyses in which the probands of the selected families carried only the DRB1^*^03:01/X (X≠04:01)* or* DRB1^*^04:01/X (X≠03:01) genotype. Changes in the LOD score profiles in these different subgroups can reflect interaction of that allele with loci linked to the phenotype [25].

## 3. Results

### 3.1. Linkage Analysis with “Any Complication” as the Phenotype, (Figure 1(a))

**Figure 1:**
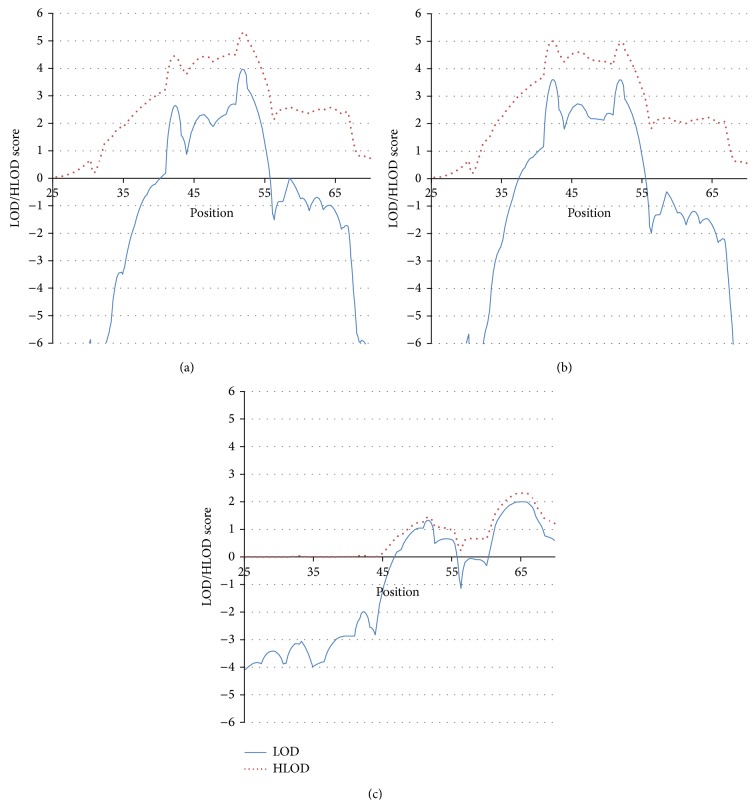
(a) Linkage analysis with “any complication” as the phenotype. (b) Linkage analysis with retinopathy as the phenotype. (c) Linkage analysis with nephropathy as the phenotype.

With the affected phenotype defined as “presence of any complication,” a large linkage peak emerged centered in the HLA region (50–52 cM); the LOD and HLOD scores at 52 cM (HLA region location) were 4.0 and 5.3, respectively. Two separate, novel loci for complications were located* outside* the HLA region (Table 4, Figure 1(a)), one telomeric (42 cM) and one centromeric (64 cM) to the HLA region. At an assumed penetrance of 0.25, the LOD was 2.6 at the 42 cM peak; the HLOD was 4.4. The LOD score at the centromeric region (64 cM) was negative, but the HLOD score was 2.6 (Table 4, Figure 1(a)), suggesting linkage in only a subset of the families.

### 3.2. Linkage Analysis with Retinopathy as the Phenotype (RET), (Figure 1(b))

RET was the most common complication found in our dataset. We saw only minor differences between the ANY COMPLICATION and RET analyses. The maximum scores at the 42 cM peak for RET were LOD = 3.6 and HLOD = 5.0 (for ANY COMPLICATION, the scores were LOD = 2.6 and HLOD = 4.4 (compare Figures 1(a) and 1(b), Table 4)). At the HLA locus, the scores for RET were LOD = 3.6 and HLOD = 5.0, and for ANY COMPLICATION, they were LOD = 4.0 and HLOD = 5.3. The HLOD scores at the broad peak around 64 cM were 2.2 for RET and 2.6 for ANY COMPLICATION. It is noteworthy that the LOD scores at the 42 cM peak* increased* significantly when including only RET as “affected” compared with ANY COMPLICATION, despite the drop in sample size, that is, excluding nephropathy and neuropathy cases. This suggests that the 42 cM peak does not contribute to the expression of nephropathy or neuropathy.

### 3.3. Linkage Analysis with Nephropathy as the Phenotype (NEPH), (Figure 1(c))

The linkage results in the 45 NEPH families show 2 peaks: the first peak occurs over the HLA region at 52 cM (LOD = 1.3 and HLOD = 1.4 (Figure 1(c))). The second peak is located at the same position (64 cM) as the centromeric peak seen in the ANY COMPLICATION and RET analyses. There is evidence* against* linkage with the NEPH phenotype at the 42 cM locus that showed strong linkage evidence for RET and ANY COMPLICATION. Furthermore, with the NEPH phenotype, the 64 cM peak shows virtually no evidence of heterogeneity (similar LOD and HLOD values) (LOD = 2.0 and HLOD = 2.2), suggesting that it is a locus uniquely linked with NEPH. This suggests that the 42 cM locus is unique to RET and does not influence NEPH.

These results reveal two novel loci that contribute to the expression of complications. These two loci appear to have differential influences on RET and NEPH: one influencing mostly NEPH (64 cM) and the other influencing only RET (42 cM). We then investigated possible interaction of these loci with HLA allele.

### 3.4. Stratification Analyses with Retinopathy as the Phenotype

#### 3.4.1. DRB1^*^03:01 Stratification (DRB1^*^03:01/X), (Figure 2(a))

We previously showed [10] that the presence of DRB1^*^03:01 reduced the risk for retinopathy while DRB1^*^04:01 increased the risk. Therefore, we repeated the current analysis subsetting out the RET families in which the proband (a) carried the DRB1^*^03:01 allele, including those with the 03:01/04:01 genotype and (b) subsetting out those that carried the DRB1^*^03:01 allele, but excluding those with the 03:01/04:01 genotype (“pure” DRB1^*^03:01). We did the two analyses to disentangle the possibly opposite effects on complications of the two different HLA-DRB1 alleles seen in our previous association analysis.

At the 42 cM locus, the unstratified RET analysis (above) had obtained a LOD = 3.6 and an HLOD = 5.0. The DRB1^*^03:01 stratification analysis still showed strong evidence for linkage, but the LOD and HLOD scores decreased (LOD = 3.0 and HLOD = 3.9), the decrease suggesting only that some families contributing to disease expression have been removed from the data. However, at the 64 cM locus, there was a significant* increase* in linkage evidence with stratification (LOD = 3.1 and HLOD = 3.4) on DRB1^*^03:01 (unstratified analysis was LOD = −1.5 and HLOD = 2.2). This finding suggests that the 64 cM locus may influence the expression of RET mainly in DRB1^*^03:01 carriers, suggesting an interaction between DRB1^*^03:01 and the 64 cM locus.

#### 3.4.2. “Pure” DRB1^*^03:01 Stratification (DRB1^*^03:01/X, (X≠04:01)), (Figure 2(b))

The above stratification analysis included DRB1^*^03:01 positive probands with the DRB1^*^03:01/04:01 genotype. Because our previous association analysis [10] suggested opposite effects of DRB1^*^03:01 and DRB1^*^04:01, we reanalyzed the data excluding DRB1^*^03:01/04:01 probands, representing a significant reduction in the sample size (see Table 3). The 64 cM linkage signal remained prominent in this subset with no evidence for heterogeneity (LOD = 2.0 and HLOD = 2.2), despite the reduced sample size. In contrast, the peak located at 42 cM decreased substantially with stratification: LOD = 0.9 and HLOD = 1.6 (unstratified: LOD = 3.6 and HLOD = 5.0), supporting the “protective” effect of DRB1^*^03:01 on RET.

#### 3.4.3. DRB1^*^04:01 Stratification (DRB1^*^04:01/X), (Figure 2(c))

We analyzed the RET data using only families of probands carrying the DRB1^*^04:01 allele, including DRB1^*^03:03/DRB1^*^04:01 heterozygotes. The LOD and HLOD scores at the 42 cM locus in the DRB1^*^04:01-stratified analysis remained high (4.1 and 4.1, resp.). This suggests that heterogeneous loci contributing to RET were eliminated in the stratified sample (hence the increase in the LOD score) but some families contributing to the LOD score were also eliminated (thus the decrease in the HLOD). At the 64 cM locus, the HLOD decreased (HLOD = 0.9) compared to the unstratified analysis (HLOD = 2.2); the LOD scores remained negative (unstratified = −1.5 and stratified = −0.5). This is in sharp contrast to the DRB1^*^03:01 stratification findings, in which the evidence for linkage at the 64 cM locus was notably stronger than in the unstratified analysis. This suggests that the 64 cM locus interacts with the DRB1^*^03:01 allele to foster the expression of RET but that it does not interact with the DRB1^*^04:01 allele. This conclusion is strengthened when we look at the results of the “pure” DRB1^*^04:01 family analysis (see below).

#### 3.4.4. “Pure” DRB1^*^04:01 Stratification (DRB1^*^04:01/X, (X≠03:01)), (Figure 2(d))

When including only DRB1^*^04:01/X, (X≠03:01) families, the signal at the 42 cM location remains notable with no evidence of heterogeneity (LOD = 2.5 and HLOD = 2.5), despite the large drop in sample size (see Table 3). At the 64 cM locus, the evidence is against linkage (LOD = −1.0 and HLOD = 0.0).

The stratification analysis results suggest that the 42 cM and the 64 cM loci interact epistatically and differentially with the DRB1^*^03:01 and ^*^04:01 alleles, revealing evidence of locus heterogeneity for each phenotype. The unstratified analysis of the RET families at the 42 cM locus yields a LOD score of 3.6 and an HLOD of 5.0, indicating substantial locus heterogeneity in the data. Analysis of “pure” 03:01 families yields a LOD < 1 for RET at the 42 cM locus and an HLOD that still suggests heterogeneity (HLOD = 1.6). The “pure” 04:01 families yield a LOD = HLOD = 2.5 at the 42 cM locus. These results suggest that the 42 cM locus interacts positively with the 04:01 allele (to produce RET) and negatively with the 03:01 allele (to protect against RET). Furthermore, when only the 04:01 allele is present (and not 03:01), the 42 cM locus shows no evidence of heterogeneity.

The DRB1^*^03:01 stratification analyses suggest that the 42 cM locus influences RET less when ^*^03:01 is present than when ^*^04:01 is present. This is expected if ^*^03:01 “protects” against RET. Like the 42 cM locus, the 64 cM locus shows evidence of interaction, but with the ^*^03:01 allele. When stratifying on the ^*^04:01 allele, there is almost no evidence for linkage at the 64 cM locus.

## 4. Discussion

In this study, we used, for the first time, LOD score linkage analysis to identify loci that contribute to the expression of the microvascular complications of RET and NEPH. Linkage analysis has been shown to have the most power to detect loci important for disease expression and has the greatest ability to give us information about the genetic characteristics of the phenotype and the existence of heterogeneity [22].

The results of the “any complication” phenotype indicated the existence of three loci. The fact that the HLA locus appeared is unsurprising [10, 26–31] but the discovery of two novel, complications-related loci at 42 cM and at 64 cM was unexpected. There was also significant evidence for the interaction of these two novel loci with the alleles DRB1^*^03:01 and ^*^04:01.

Statistically significant evidence for the existence of two loci for complications adds strong support for inherited influences on complications' expression. The two loci appear to affect RET and NEPH differently. Both appeared to influence RET expression but the 64 cM locus appeared to influence only NEPH and the 42 cM locus had no influence on NEPH.

The significant change in the 64 cM locus's LOD score among proband families with DRB1^*^03:01 strongly suggests that the presence of DRB1^*^03:01 increases the influence of the 64 cM locus on RET expression. The influence of the 64 cM locus virtually disappears when the proband has the 04:01 allele while the 42 cM peak is strengthened. When proband families are not selected for having a particular HLA allele, the observed evidence for heterogeneity is expected if the two HLA alleles contribute differentially to the phenotype. The positive differences in the LOD scores between the stratified and unstratified samples are strong indicators of interaction of the HLA alleles with the two loci [32].

The strong linkage evidence at the HLA region (52 cM) might indicate the influence of HLA on complications (known from association evidence) or merely cosegregation of HLA alleles with diabetes in general regardless of complications. Changes in the HLA linkage profile under stratification are not interpretable because we artificially altered the HLA allele structure by including or excluding specific alleles. However, finding that the DRB1^*^03:01 and ^*^04:01 alleles interact with the novel loci confirms that HLA influences complications' expression.

This study is not without ambiguities.

1. The linkage region we have identified (30 cM–70 cM) is a relatively small one for most linkage analyses; yet we have observed three distinct loci with specific effects. One of those loci is the HLA region, which strongly affects T1D expression. Were we analyzing the T1D phenotype and not complications, the LOD score at HLA would be on the order of 40, thus swamping any other T1D-related signals. However, the narrowness of the region does not nullify the clear separateness of the linkage signals. No matter how the data are stratified and broken down, the consistency of the 42 cM and 64 cM peaks, even when the 42 cM peak disappears (as in the analysis of NEPH), argues strongly that these loci influence complications' expression.
2. The number of linkage analyses that we have performed may lead to the question as to whether the LOD scores for the two loci we identified should be subject to correction for genome-wide testing. All three peaks appear in the first analysis with notable (2.5–5) LOD scores and/or HLOD scores and the locations of these peaks were invariant. The information content of the genotypic data did not fall below 98% across the region. The usual criterion for significance of a LOD score (variously debated to be from 2.5 to 4.0) is for* genome-wide* significance, that is, examining marker loci over the entire* genome*. However, we examined only markers on chromosome 6, which constitutes only about 6% of the genome and, therefore, the genome-wide cut-off values are too conservative for this analysis. Even so, several of the peaks did reach genome-wide significance levels, which became even higher under stratification (i.e., with a smaller dataset). We did not attempt to correct LOD scores under stratification because the stratification hypothesis did not concern the* existence* of a peak but how it changed under stratification. Some of those changes would have to be viewed as statistically significant indications of interaction [32].
3. We used changes in the height of the peaks as indicators of the loci's influence on complications' expression. The question of the relative strengths of influence on gene expression as related to linkage peak height is not a well-studied area. Linkage will most likely only be observed with loci “necessary” for disease expression [33]. Using the relative LOD score changes as indicators of heterogeneity and of interaction is an expansive use of linkage analysis that has been applied to good effect in our studies of autoimmune thyroid disease [34] as well as other diseases [35]. The information inherent in linkage analysis is extremely rich and can be exploited to learn about heterogeneity, mode of inheritance, pleiotropy, and gene-gene interaction.
4. Previous work has demonstrated how the stratification technique we used can identify epistatically interacting loci [25]. Recent work [32] on detecting interaction has shown that epistasis is easily differentiated from heterogeneity and that false positive indications of interaction are unlikely. However, we cannot yet quantify the degree of interaction based on changes in the LOD score. Also, because of sample size changes, changes in LOD scores cannot always be unambiguously assigned to changes in interaction. However, an increase in the LOD score that accompanies a decrease in the sample size argues for a “purification” of the sample. Thus, an increase based on stratification is a clear indicator of interaction.

The next step in this work is to analyze the whole genome, now that we know the importance of stratification loci in identifying interaction. While applying this study's stratification approach may help us identify the specific genes in the linkage regions using association analysis of SNPs with retinopathy and/or nephropathy, the option also now exists to use next-generation sequencing to identify the disease-related variants. The difficulty, as with other common conditions, is identifying the disease-related variant if such variants do not occur in exons.

Replicating our work in other samples is highly desirable. However, since the wide adoption of GWAS as the genetic technique of choice and the accompanying decrease in the collection of family data, it is not clear how much family data exist for linkage of complications. Nevertheless, family studies are the best way to effectively use the newest genetic technologies [36], and we hope that our findings will inspire a resurgence of family studies for T1D complications and the search for heterogeneity.

## Figures and Tables

**Figure 2:**
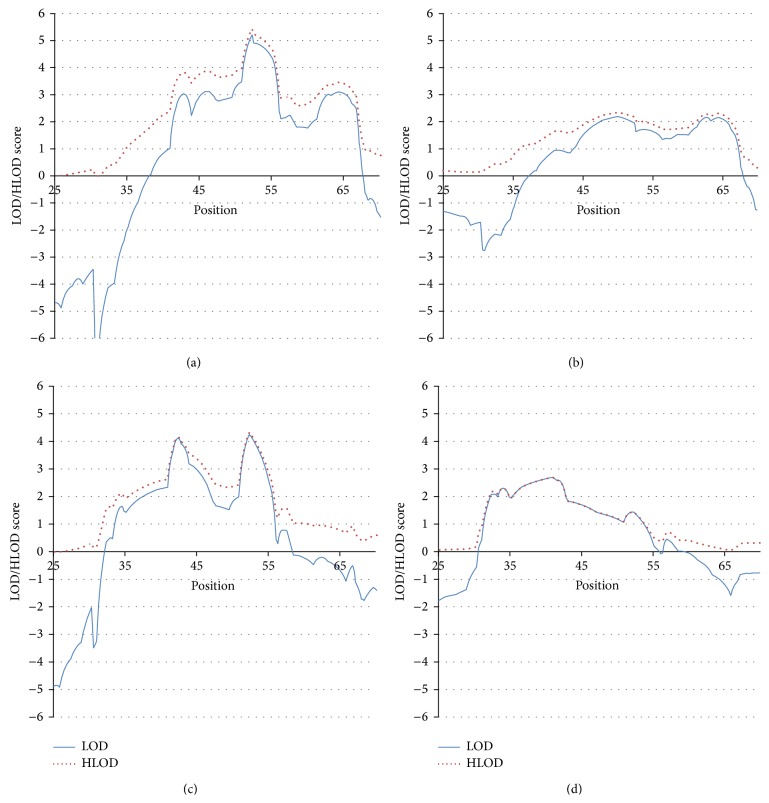
(a) Linkage analysis with retinopathy as the phenotype, stratified by families whose probands are positive for DRB1^*^03:01/X = any allele. (b) Linkage analysis with retinopathy as the phenotype, stratified by families whose probands are positive for DRB1^*^03:01/X, where X≠DRB1^*^04:01. (c) Linkage analysis with retinopathy as the phenotype, stratified by families whose probands are positive for DRB1^*^04:01/X. (d) Linkage analysis with retinopathy as the phenotype, stratified by families whose probands are positive for DRB1^*^04:01/X = any allele, where X≠DRB1^*^03:01.

**Table 1:** Number of families with affected (T1D + complications)-unaffected (T1D only) members.

Affected-unaffected family members	*N*_families_ (%)
1 affected-1 unaffected	68 (16)
2 affected-0 unaffected	50 (12)
0 affected-2 unaffected	210 (51)
Other	87 (21)

Total	415 (100)

**Table 2:** Prevalence of clinical characteristics among 415 T1D families.

Clinical characteristic	Number (%) of individuals
Total	2,008 (100.0)
T1D + microvascular complications	239 (11.9)
T1D + retinopathy	219 (91.6)
T1D + nephropathy	87 (36.4)
T1D + neuropathy	76 (31.8)
T1D only	629 (31.3)
No T1D	1140 (56.8)

**Table 3:** LOD score summary table.

LOD (HLOD) scores			
Phenotype	42 cM peak	52 cM peak	64 cM peak
Any complication	2.6 (4.4)	4.0 (5.3)	−1 (2.6)
Retinopathy	3.6 (5.0)	3.6 (5.0)	−1.5 (2.2)
Nephropathy	−2.0 (0.0)	1.3 (1.4)	2.0 (2.2)
Retinopathy + nephropathy analyzed together	3.2 (4.8)	4.0 (5.2)	−1.1 (2.3)

Stratification (retinopathy only)			
DRB1^*^03:01	3.0 (3.9)	5.1 (5.3)	3.1 (3.4)
DRB1^*^04:01	4.1 (4.1)	4.2 (4.2)	−0.5 (0.9)
DRB1^*^03:01/X	0.9 (1.6)	2.0 (2.2)	2.0 (2.2)
(X≠DRB1^*^04:01)			
DRB1^*^04:01/X	2.5 (2.5)	1.4 (1.4)	−1.0 (0.0)
(X≠DRB1^*^03:01)			

Numbers in parentheses are HLODs.

**Table 4:** Numbers of families in the subgroups.

Complication	Stratification subgroup	Number of families included	Count of people included
Any complication		159 families	1015 people

Retinopathy	All families	144 families	928 people
	DRB1^*^03:01	61 families	409 people
	DRB1^*^04:01	58 families	336 people
	Pure DRB1^*^03:01	37 families	266 people
	Pure DRB1^*^04:01	35 families	199 people

Nephropathy		45 families	325 people
